# Who’s afraid of synthetic data? Hybrid approaches to deliver medical digital twins

**DOI:** 10.1016/j.imu.2026.101737

**Published:** 2026-01-19

**Authors:** Joel Vanin, Amit Hagar, James A. Glazier

**Affiliations:** a Department of Intelligent Systems Engineering, Indiana University, Bloomington, IN, USA; b Biocomplexity Institute, Indiana University, Bloomington, IN, USA; c Department of History and Philosophy of Science and Medicine, Indiana University, Bloomington, IN, USA

**Keywords:** Synthetic data, Digital twins, Virtual tissue models, Multiscale mechanistic modelling, Machine learning, Precision medicine, Model validation, Algorithmic bias

## Abstract

Despite rapidly growing volumes of clinical data, precision medicine still faces a structural data deficit: most patients and rare disease variants are sparsely sampled, labels are noisy, and counterfactual outcomes for alternative treatments are fundamentally unobservable. This position paper argues that overcoming these limits will require hybrid systems that couple multiscale virtual tissue models, synthetic data generation, and AI/ML within risk-aware digital twin frameworks. Using a structured narrative synthesis of three literatures—synthetic health data, virtual tissues and medical digital twins, and hybrid mechanistic–AI architectures including numerical weather prediction—we develop a conceptual framework centered on a mechanistic core linked to AI via forward (mechanistic → synthetic data → AI), backward (AI → mechanistic), and closed (patient-anchored digital twin) loops. We analyze how complex-systems behavior, biological adaptability, and sparse observations bound what medical digital twins can meaningfully predict, motivating ensemble and population-level forecasts rather than exact individual replicas. We then survey emerging implementation patterns, parameter-space exploration methods, and computational envelopes for using virtual tissues to generate biologically constrained synthetic cohorts and to calibrate hybrid digital twins. Finally, we adapt risk- and context-informed verification, validation, and governance frameworks to a four-layer stack spanning mechanistic cores, synthetic data products, AI components, and clinical workflows, with explicit attention to bias, drift, and provenance. We conclude that near-term impact is most likely from population- and cohort-level digital twins that support stratification and short-horizon decision support, while laying the groundwork for more individualized, trustworthy hybrids as biological and methodological uncertainties are better characterized.

## 1. Introduction – Overcoming data barriers: the role of synthetic data and digital twins

The promise of precision medicine depends on data that are abundant, representative, well-labelled, and longitudinal. In practice, clinical datasets rarely meet these conditions. Rare diseases and atypical phenotypes are systematically under-sampled; routine care generates fragmented records with inconsistent follow-up; and regulatory, legal, and logistical barriers limit reuse and sharing of high-value data.

Two technological lines of work have emerged as complementary responses to these constraints. Synthetic data generation uses algorithms to create artificial datasets that mimic the statistical structure of real clinical data while reducing privacy risk and facilitating data sharing [1–3]. In parallel, digital twins have evolved from engineering and manufacturing into health, where they are envisioned as dynamic computational replicas of patients, organs, or processes that are repeatedly updated with new data [4–9].

Within this landscape, multiscale, physics-based virtual tissue models occupy a distinctive niche. By encoding biophysical processes into executable simulations, they act as mechanistic “engines” for digital twins and as generators of biologically constrained synthetic cohorts [10–12]. When used to generate synthetic cohorts, these models transform decades of experimental and clinical knowledge into richly annotated, biologically constrained datasets—images, time series, and trajectories—that can be consumed by machine learning systems [13, 14].

In this review, we argue that the most productive path forward is to treat synthetic data, virtual tissues, digital twins, and AI as components of a single hybrid ecosystem rather than as siloed technologies. Section 1.1 examines the specific limitations of real-world clinical data that motivate synthetic and mechanistic approaches. Section 1.2 situates virtual tissues within the broader digital-twin paradigm. Section 1.3 briefly describes how we identified and synthesized the literature, and Section 1.4 introduces a conceptual framework that organizes the rest of the review.

### 1.1. The challenge of real-world clinical data

Recent reviews of synthetic data in healthcare and rare disease research emphasize that small cohorts, heterogeneous phenotypes, and privacy constraints remain central obstacles for both traditional statistics and modern machine learning [1–3,15]. The result is a data paradox: precisely the patient groups and clinical questions for which we most need robust predictions are those for which real-world data are most limited. Beyond simple scarcity, two intertwined limitations dominate: (i) data-quality problems, where labels and measurements are noisy or inconsistent, and (ii) structural features of clinical research design that prevent observation of many outcomes most relevant for precision medicine.

On the *data quality* side, clinical settings struggle to establish robust ground truth. Diagnoses, assessments of disease progression, and treatment responses are often inferred indirectly rather than measured directly, creating substantial label uncertainty. Even in apparently standardized domains such as digital pathology, systematic reviews document nontrivial inter-observer disagreement and variability in diagnostic criteria [16–18]. AI systems can, in principle, standardize criteria and reduce inter-observer variability [16,18], but their performance depends on training datasets with clear and consistent labels. This creates a circular dependence: high-quality labels are needed to build the tools that would improve labelling, limiting the reliability of purely data-driven approaches [19].

*Structural constraints* impose deeper limitations often described as a counterfactual gap. Randomized controlled trials estimate average treatment effects across populations, but each patient follows only one arm; it is impossible to observe what would have happened to the same individual under alternative sequences, doses, or timing. Clinical datasets thus trace only one branch of a larger tree of potential trajectories. Large-scale disease-trajectory studies show that even rich longitudinal datasets capture only a subset of possible paths through “disease space” [20], making it difficult for AI models trained on population averages to predict individualized responses in heterogeneous diseases where tumor evolution, immune dynamics, or co-morbidities create patient-specific courses. Crucially, these limitations cannot be removed by collecting more observational data: the unobserved counterfactuals are missing by construction. Virtual tissue modelling directly targets this gap by enabling virtually controlled *in silico* experiments that explore alternative trajectories for the same “virtual patient” while maintaining biological fidelity [10,21,22].

### 1.2. The digital twins paradigm in biomedicine

Digital twins originated in engineering for real-time monitoring and optimization of physical systems through continuous sensor feedback and predictive modeling [4–6]. They have been deployed for aerospace vehicles, manufacturing, and urban systems, where dense sensor networks and well-understood physics enable tightly coupled virtual and physical systems [23]. Translating this paradigm to biomedicine is attractive but challenging [7–9].

*Healthcare digital twins* aim to create continuously updated, patient-specific models that integrate wearable sensors, periodic clinical measurements, and electronic health record data, including imaging, multiomics, and physiological signals [8,9]. Such systems could, in principle, support virtual intervention testing, early disease detection, and treatment optimization by comparing realized and simulated outcomes. However, biological systems pose distinct challenges: many biomarkers are difficult to measure continuously, key processes span multiple scales from molecular to organ level, and the underlying regulatory networks can change under therapy [12,24]. Scoping reviews conclude that fully dynamic, real-time clinical twins remain aspirational; most existing systems are preclinical, proof-of-concept, or limited to narrow use cases [8].

Within this emerging paradigm, *virtual tissue models* provide organ- and microenvironment-scale mechanistic cores. Cell-based and multiscale frameworks such as CompuCell3D and PhysiCell capture how local rules—cell adhesion, proliferation, migration, and death—combine with diffusing fields and tissue mechanics to generate emergent tissue-scale behaviors [10–13,25,26]. When coupled to clinical or experimental data, these models can be parameterized to represent classes of patients or specific individuals and used to generate synthetic cohorts that explore plausible trajectories and treatment responses [21,22,27]. In this sense, synthetic data from virtual tissues function less as generic “fake data” and more as a form of knowledge compression, unpacking mechanistic assumptions into observable patterns that would be infeasible or unethical to sample exhaustively *in vivo*. At the same time, work on integrating machine learning and multiscale modelling has articulated general patterns for using AI as a surrogate, parameter inference engine, or model-reduction tool within such systems [24,28,29]. Together, these developments suggest viewing digital twins not as isolated AI systems but as hybrid assemblies in which mechanistic models, synthetic data generators, and data-driven components play complementary roles.

### 1.3. Position, scope, and literature selection

This position paper analyzes critical gaps in our current ability to deliver medical digital twins and argues—drawing on lessons from numerical weather prediction—that addressing these gaps will require extensive use of synthetic data and hybrid architectures that combine multiscale mechanistic models with data-driven AI/ML components. As part of this argument, we briefly review key obstacles to medical digital twins, survey the state of synthetic data and hybrid approaches in other domains, and propose a four-layer, risk-informed framework for validation, bias analysis, and governance of hybrid virtual-tissue–AI systems. We close by outlining a roadmap for near-term and longer-term research priorities.

Our argument is grounded in a structured narrative synthesis of three partially overlapping literatures rather than in a formal systematic review: (i) synthetic data generation in healthcare and rare-disease research, (ii) digital twins and multiscale virtual tissue modelling in biomedicine, and (iii) hybrid mechanistic–AI architectures, including weather and climate forecasting. We identified publications through iterative keyword searches in PubMed and Google Scholar, targeted arXiv/bioRxiv searches for fast-moving areas (e.g., diffusion-based generative models, ML-based weather forecasting), and citation chaining from recent reviews of synthetic data in healthcare [1–3,15] and medical digital twins [4,8,9]. We prioritized peer-reviewed English--language articles published between 2020 and December 2024, supplemented by earlier foundational work in complex systems and atmospheric modelling where needed for conceptual background. Our aim is not an exhaustive catalogue, but a curated set of exemplars that concretely instantiate the patterns and governance issues we highlight.

### 1.4. Conceptual framework

**Fig. 1.**
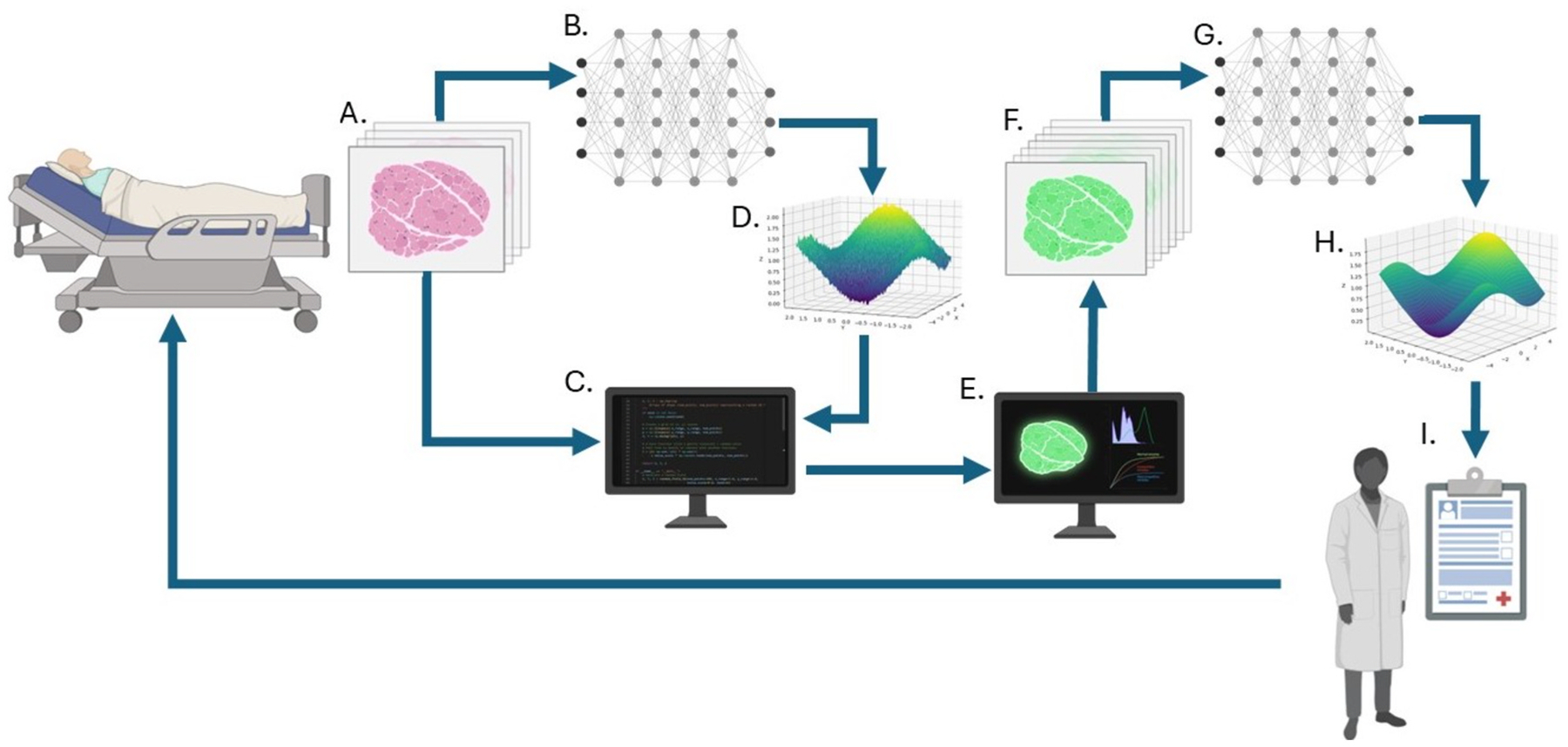
Integrated framework for synthetic data generation and AI-driven diagnostics in clinical applications. Schematic overview of a bidirectional framework linking patient data, AI models, and agent-based or multiscale virtual tissues. Clinical data (A) are used both to train initial AI/deep neural network (DNN) classifiers (B) and to parameterize mechanistic models using clinical and literature-derived parameters (C). AI training defines a knowledge manifold with characteristic noise patterns that can constrain mechanistic parameters (D). Virtual tissue simulations generate *in silico* experiments and quantitative metrics (E), which are used to synthesize data across disease states and time (F). Real and synthetic data are then combined for secondary AI/DNN training (G), refining the knowledge manifold and improving classification and generalization (H). Model outputs are presented through a clinical decision-support interface (I) that provides probabilistic forecasts of disease progression and treatment response, feeding back into patient monitoring and treatment adjustment and thereby closing the digital-twin loop. Created in BioRender. Vanin, J. (2025) https://BioRender.com/c43d769.

To organize the diverse literature, we adopt a framework that treats virtual tissues, synthetic data, digital twins, and AI/ML as components of a coupled system rather than isolated technologies (Fig. 1). At its center is a mechanistic core consisting of multiscale, physics-based virtual tissue or organ-level models parameterized to represent classes of patients or specific individuals [10–12]. Around this core we distinguish three interactions (“loops”) and a cross-cutting validation layer: (i) a forward loop from mechanistic models to synthetic data and AI, (ii) a backward loop from AI to mechanistic models, and (iii) a closed loop in which the combined system functions as a digital twin anchored to a patient or cohort.

#### Forward loop: mechanistic models → synthetic data → AI.

Virtual tissue simulations can generate synthetic cohorts of “virtual patients” by sampling biologically plausible parameter ranges and initial conditions, producing annotated images, time series, and outcome trajectories. Image-driven pipelines that convert 3D microscopy or spheroid images into executable agent-based models illustrate how real data can initialize such simulations [21,27,30], and large virtual patient ensembles have been used to explore tumor–immune dynamics and treatment responses *in silico* [22]. These synthetic datasets can then be used to train or stress-test AI models—classifiers, segmentation networks, or reinforcement learning policies—particularly in regimes where real clinical data are sparse or biased, such as early disease progression, rare phenotypes, or atypical treatment courses [1,15,31,32]. In this loop, synthetic data function as a controlled, mechanistically grounded augmentation of real-world data rather than as a replacement.

#### Backward loop: AI → mechanistic models.

Data-driven methods can act as surrogates for expensive simulations, accelerate parameter exploration, or infer latent parameters and structures that reconcile model predictions with observed data. Alber et al. (2019) outline patterns for integrating multiscale modelling with machine learning, including parameter identification, model reduction, and closure relations [28]. Recent work on mechanistic learning in oncology combines mechanistic ODE/PDE or agent-based models with neural components and manifold-learning approaches to improve fit to experimental and clinical data [29,33,34]. In our framework, such approaches allow clinical and experimental data to push back on mechanistic assumptions in a principled way: discrepancies between model outputs and data become signals to refine model structure, constrain parameter ranges, or propose new biological hypotheses, rather than being dismissed as noise.

#### Closed loop: digital twins anchored to patients.

In the outer loop, mechanistic and AI components are jointly calibrated to longitudinal data from an individual patient or from a defined cohort—imaging, laboratory values, and other clinical signals—and then used to simulate alternative intervention strategies, predict near-term trajectories, or guide adaptive therapy [8,9,22]. Work on patient-specific virtual tissues, model-informed deep-learning–based adaptive therapy, and reinforcement-learning–guided dosing illustrates early forms of such closed loops in oncology [35,36]. Synthetic trajectories generated by the twin are continually compared with realized outcomes; significant discrepancies trigger recalibration of both mechanistic and AI components.

#### Validation and governance as cross-cutting layers.

All three loops must operate within explicit validation and governance structures. Synthetic data and model predictions are not assumed to be “true” by construction: their credibility depends on verification and validation of the mechanistic core, quantitative evaluation of synthetic datasets, and rigorous testing of AI models on independent real-world data, all tailored to clearly defined contexts of use and risk levels [12,37–40].

Fig. 1 thus serves as a roadmap for the remainder of the article. Section 2 develops the theoretical underpinnings of the mechanistic core and its relationship to synthetic data, including the weather-forecasting analogy. Section 3 uses the forward, backward, and closed loops to organize concrete architectural patterns and examples for implementations. Section 4 focuses on validation, bias, and governance challenges for these hybrid systems.

## 2. Theoretical foundations of synthetic data in biological systems

### 2.1. Complex systems theory in biological data

Biological tissues are complex adaptive systems (CAS): many interacting components generate emergent behavior across scales, from genes to organs, in ways that cannot be reduced to any single level of description [41–43]. This multiscale structure is not an artifact of missing information, but a fundamental property of living systems. Pereira and colleagues, for example, show that mapping a single tissue sample requires bridging millimeter-scale architecture and nanometer-scale subcellular organization, each with distinct organizational principles [44]. For synthetic data generators and digital twins, this means that useful models must encode both local interactions and higher-level constraints rather than focusing on a single “privileged” scale.

As living systems, tissues also maintain homeostasis far from equilibrium and actively exploit stochasticity instead of treating it as mere noise [41,45]. Fluctuations in gene expression, signaling, and cell–cell interactions produce branching “cones” of possible futures rather than a single deterministic trajectory for any individual. At the population level, this motivates ensemble simulations and probabilistic forecasts; at the individual level, it limits the granularity with which we can meaningfully predict future states [46]. In the context of synthetic data and digital twins, the practical implication is that models should be designed to generate ensembles of plausible trajectories and variability patterns, and validation should focus on whether distributions, regime changes, and rare-but-plausible behaviors are captured, rather than on exact pointwise agreement with any one time series [30]. Section 2.5 returns to how these constraints define the epistemic limits of prediction and appropriate contexts of use.

### 2.2. Virtual tissue modelling: Mechanistic approaches

Within this complex-systems perspective, virtual tissue models provide a mechanistic substrate for synthetic data generation. They represent tissues or organs as coupled dynamical systems linking intracellular regulation, cell behaviors, and tissue-scale transport or mechanics, typically by combining compartmental ODEs, agent-based rules, and continuum PDE descriptions [10–12]. Rather than reproducing every molecular detail, these models encode the processes believed to drive emergent structure and dynamics, and recent reviews offer detailed taxonomies of frameworks in oncology, immunology, and regenerative medicine [13,14,25,26,47,48].

Once calibrated, virtual tissues can generate synthetic datasets that are difficult or impossible to obtain *in vivo*. By systematically varying parameters such as cell-cycle duration, adhesion strengths, or mutation patterns, they can simulate rare phenotypes, early disease stages, and counterfactual treatment strategies for the same “virtual patient,” including combinations of surgery, radiation, and systemic therapies [22,35,36]. Simulation outputs span temporal traces, cell-population dynamics, and spatial organization. When rendered through virtual histology or virtual staining pipelines, they yield synthetic microscopy tiles that preserve realistic morphology and staining artefacts, which have been used to augment diagnostic training sets and stress-test AI algorithms on controlled distributions of disease states [32,49]. Calibration and validation against independent imaging, multiplexed tissue measurements, and longitudinal outcomes are central to their credibility as synthetic data generators; these issues reappear in Sections 2.4 and 4, where we discuss data–model integration and multi-layer validation in more detail [14,30].

### 2.3. Weather forecasting analogy: Lessons and limits

Weather forecasting is often used as a metaphor for hybrid mechanistic–AI systems, and it offers both inspiration and warnings. Early numerical weather prediction faced constraints similar to current virtual-tissue modelling: limited computational resources forced coarse grids and simplified physics, so models had to balance resolution, complexity, and run time to deliver forecasts faster than real time [50]. Over time, progress came from combining three strands—physical models, statistical and empirical models, and hybrid approaches—each tuned to different questions and horizons, from short-term local weather to longer-term climate patterns [50,51].

Recent machine-learning weather systems such as FuXi, GraphCast, FourCastNet, and related probabilistic forecasters are trained on multi-decadal reanalyses like ERA5 and now match or surpass leading NWP models on many global medium-range metrics, often at much lower computational cost [52–57]. These systems do not replace physics; instead, physics-based models and ML forecasters are increasingly used in tandem, with hybrids exploiting the strengths of each.

The analogy is powerful but limited. The atmosphere obeys fixed physical laws, whereas biological systems can change their effective rules via evolution, plasticity, and therapy-induced adaptation [58]. Meteorology benefits from dense, standardized, near-global datasets (e. g., ERA5), while biomedicine has fragmented, biased, and sparse data distributions with no equivalent to a long, homogeneous “ERA5 of human disease” [8,20,52]. Weather forecasts are largely non--reflexive—the forecast does not alter the dynamics—whereas clinical predictions can change treatment and thus the data-generating process. Ethical and practical constraints also limit sensorization and experimentation in patients in ways that do not apply to the atmosphere.

Taken together, the weather analogy suggests *how* to combine mechanistic and data-driven models, but not that biology can be forecast with similar accuracy. For virtual tissues, synthetic data, and digital twins, the key lessons are to treat predictions as ensembles of plausible trajectories rather than single point forecasts, to match model architecture and resolution to specific questions and horizons, and to continually re-anchor models to new data rather than assuming stationarity. These themes reappear when we discuss contexts of use, uncertainty, and validation in Sections 3 and 4.

### 2.4. Bridging virtual and real data in biological systems

Bridging virtual tissues and real biology requires explicit pipelines that connect imaging, omics, and clinical measurements to model states and parameters, while respecting the adaptive, non-stationary nature of living systems. Unlike atmospheric physics, biological regulation can rewire under selection and therapy, and microenvironmental signals, microbiota, and mechanical forces continuously reshape gene expression and metabolism [41,45,59,60]. Multiscale models must therefore be calibrated and validated with data that probe both structure and dynamics across scales.

Recent work illustrates concrete strategies. Nürnberg et al. (2024) [27] use segmented 3D confocal microscopy of cancer spheroids to initialize Cellular Potts simulations in CompuCell3D and define morphological metrics that guide parameter tuning so simulated spheroids match experimental structure. Yue and Dutta (2022) [34] construct multi-layer network models linking genomic, proteomic, and metabolomic data to cellular and tissue phenotypes, providing a route to embed pathway-level knowledge into higher-scale dynamics. Multiplexed imaging platforms such as CODEX add another layer by measuring dozens of protein markers while preserving spatial context, enabling stringent tests of model-predicted spatial patterns and cell-type distributions at specific time points [30].

These examples highlight that different data types play distinct roles in the model lifecycle. High-dimensional static data, including multiplexed imaging, are well suited for initializing model states and constraining spatial organization. Longitudinal experimental or clinical measurements test dynamic trajectories, and cohort-level data constrain outcome distributions and regime frequencies [61]. The “curse of the cartographers” [62] reminds us that a single model cannot reproduce all scales and details simultaneously: attempts at exhaustive representation can undermine interpretability and tractability. Instead, effective bridging strategies explicitly choose which scales and observables matter for a given context of use, match each data type to specific tasks (parameter inference, structural validation, or dynamic testing), and accept that some aspects of biology will remain coarsely resolved. Virtual-tissue-based synthetic data thus sit within a triangle linking mechanistic models, experiments, and clinical data, with each edge governed by its own validation and uncertainty assessments [12,61].

### 2.5. What can we actually predict? Epistemic limits and model design

Recent work on digital twins emphasizes that they should be understood as decision tools, not perfect virtual replicas of individual patients. The National Academies define a digital twin as a virtual construct that mimics a system, is dynamically updated with data from its physical counterpart, has predictive capability, and *informs decisions* via a bidirectional feedback loop between model and reality [63]. In this framing, the goal of a biomedical digital twin is to provide calibrated, decision-focused predictions whose scope and limits are explicitly characterized for particular questions, horizons, and patient populations.

Within that framing, the preceding sections and results from several fields point to hard limits on what any hybrid mechanistic–AI system can deliver. In ecology and population dynamics, model-free or low-parameter approaches can outperform even the “correct” mechanistic model at short forecast horizons, and all models eventually lose skill as horizon lengthens [51,64,65]. Malmborg et al. (2024) [66] similarly find that forecasts built on aggregated, coarser-scale variables are often more robust than those based on fine-grained states, because small-scale fluctuations are both noisy and weakly observed. In oncology, Yu and Bagheri (2024) [67] show that increasing mechanistic complexity in spatial cancer models does not guarantee better insight or out-of-sample performance; detailed biology is valuable where it is constrained by data and relevant to the decision, but beyond that envelope it mainly adds parameters and identifiability problems. Tumor evolution, immune escape, and therapy-induced plasticity mean that models calibrated today may be systematically mis-specified tomorrow, motivating adaptive-therapy and mechanistic-learning frameworks that explicitly treat evolution and treatment as coupled processes to be modelled and optimized [29,35,36,58].

These insights translate into concrete design principles. Questions and forecast horizons must be specified up front. For example, a model intended to choose between two chemotherapy schedules over six months has different structural and data requirements than one used to explore resistance mechanisms over years. Evaluation should target ensembles and uncertainty, asking whether models capture distributions, transitions, and qualitative regime changes—such as the emergence of resistant clones or shifts in immune dominance—rather than optimizing single-point error metrics. Finally, AI components are most useful when deployed as surrogates or inference engines that accelerate parameter exploration or fit mechanistic models to data, while keeping parameters interpretable and within biologically plausible ranges [28, 33,68,69].

At present, the most reliable forecasts are typically population-level. As homeostatic and disease “spaces” become more systematically mapped, digital twins for precision medicine are best viewed as tools that locate an individual within these stratified spaces and estimate probabilities of near-term transitions, rather than as exact simulators of a unique microscopic future. At the same time, AI models can discover patterns not explicitly encoded in mechanistic structures, raising questions about how to interpret and trust such patterns; advances in explainable AI and model interpretability in histopathology and related domains [70] offer tools for linking predictions back to candidate mechanisms or spatial features, but do not eliminate the need for explicit mechanistic reasoning. Taken together, these insights argue for a risk- and context-aware approach: virtual tissues and hybrid mechanistic–AI systems should be evaluated as bounded, question-specific approximations that generate ensembles of plausible trajectories and regime classifications. Synthetic data can extend and stress-test these models within well-defined domains, but cannot remove the fundamental limits imposed by biological adaptability and data constraints. This logic underpins the risk- and context-informed validation frameworks discussed in Section 4, including ASME V&V 40, emerging Good Simulation Practice, and related regulatory guidance that tie required levels of evidence to model risk, context of use, and decision impact [12,37,38].

## 3. Technical implementation of virtual tissue models for AI training

Building on the loops introduced in Section 1.4, this section summarizes how existing work instantiates these ideas in practice. Rather than proposing a single canonical architecture, we group the literature into three themes: (i) architectural patterns that realize forward, backward, and closed loops (Section 3.1); (ii) methods for exploring and constraining parameter spaces (Section 3.2); and (iii) computational and workflow considerations for deploying such systems at realistic scales (Section 3.3).

### 3.1. Emerging architectural patterns

Published systems that combine virtual tissues with AI typically implement one or more of the loops in Fig. 1 rather than a complete digital twin. Most couple (i) image-based or multimodal AI models, often in digital pathology [71–74] or radiomics, with (ii) a mechanistic simulation engine [10,11], and (iii) data pipelines that move information between components—for example, model-derived features feeding an AI [75,76], or AI-derived constraints refining the simulator [77].

In a *forward-loop* configuration, virtual tissues act primarily as synthetic-data engines. Deep networks trained on whole-slide images or other clinical modalities are augmented with simulated trajectories or “virtual histology” that cover rare phenotypes, early transition states, or atypical treatment courses that are sparsely represented in real data [31, 32,49,58]. Hybrid models that concatenate mechanistic summaries of tumor evolution with learned image features have already improved survival or response prediction relative to purely data-driven baselines [29,35,76,77]. Graph neural networks inspired by geophysical forecasting (e.g., GraphCast-style [54] architectures) extend this pattern by representing tissues as graphs whose nodes are cells and edges encode physical or signaling contacts, enabling analysis of both simulated and real tissue graphs in a shared representation [54,57].

The same simulation engines support *backward-loop* roles. Multiscale agent-based and continuum models, calibrated to imaging, blood biomarkers, or immune-panel readouts, define a structured hypothesis space over parameters such as adhesion, proliferation, death, and diffusion coefficients [12,21,22,27]. AI models then learn mappings from observables to this parameter space, enabling construction of virtual cohorts whose trajectories remain compatible with both the mechanistic rules and observed variability [36]. In practice, the simulator anchors both forward loops (synthetic cohorts and counterfactuals) and backward loops (parameter inference and model refinement).

Near-term realizations of the closed loop appear in model-informed reinforcement learning and early oncology digital-twin prototypes. Reinforcement-learning agents have been trained against mechanistic tumor-evolution or PK/PD simulators to propose adaptive dosing schedules, which are then evaluated retrospectively or in pilot clinical settings [35,78,79]. Scoping reviews of digital and virtual twins in oncology and healthcare more broadly conclude that most implementations remain preclinical or limited-deployment prototypes and that few yet satisfy all criteria for fully dynamic, continuously updated clinical twins [8,80,81]. Nonetheless, these examples show that each edge of the forward–backward–closed loop is already instantiated in concrete biomedical applications, even if full real-time twins remain aspirational.

### 3.2. Parameter space exploration for AI training

Integrating virtual tissues and AI raises two coupled parameter-space problems: generating ensembles of biologically plausible trajectories for the forward loop, and inferring mechanistic parameters or latent states from multimodal data for the backward loop. Within the forward, backward, and closed loops of Section 1.4, parameter-space exploration is supported by recurring methods (Table 1). These methods differ in how they sample, constrain, and interpret the parameter space of a virtual tissue model, but they all address the same core problem: linking mechanistic parameters and trajectories to observable clinical and experimental data.

For the *forward loop*, manifold learning and related dimensionality-reduction methods identify low-dimensional embeddings of high-dimensional dynamics, enabling more efficient sampling of parameter regimes while preserving key behaviors [33,82]. In parallel, virtual staining and tissue-synthesis methods demonstrate that generative models can produce realistic histology conditioned on underlying structure or labels, providing spatially resolved synthetic data for training and stress-testing image-based AI [32,83,90]. This demonstrates usability in regimes where real data undersample early disease progression, rare phenotypes, or atypical treatment courses.

From the *backward-loop* perspective, the central task is mapping observed imaging, omics, and clinical time series to mechanistic parameters or latent states. Bidirectional encoders and variational autoencoders can compress simulation outputs into low-dimensional latent variables and, when trained jointly on simulated and real data, learn correspondences between observed patterns and regions of parameter space. Simulation-based inference methods make this idea explicit by training neural density estimators on large ensembles of simulations to approximate the posterior over parameters given observed data; recent work shows how such approaches can be used to build virtual patients whose parameters remain consistent with both model structure and measured variability [69,84,91].

Within this backward-loop setting, Bayesian networks and symbolic-regression methods provide complementary ways to structure parameter inference. Bayesian networks offer a probabilistic graphical formalism for encoding prior biological structure and uncertainty, and have long been used to infer latent interaction networks from noisy molecular and physiological data [85,86]. Embedded in a virtual-tissue framework, they can serve as an intermediate layer linking heterogeneous observables—imaging features, laboratory values, pathway-activity scores—to latent mechanistic states or parameter clusters, making explicit which dependencies are assumed a priori and which are inferred from data. Symbolic-regression approaches such as PySR and LogicSR add an interpretable counterpart by discovering explicit equations that map a small set of features to outcomes or parameters [87,88]. In this context, they can translate transcriptomic or pathway-activity signatures into agent-based model rules—for example, mapping gene-expression patterns to division rates, death probabilities, or migration speeds—so that high-dimensional omics data enter the mechanistic model through human-readable relations rather than opaque latent vectors.

Active-learning and model-informed reinforcement-learning strategies then operationalize parameter-space exploration across both loops. Uncertainty estimates over class labels, parameter posteriors, or policy performance can be used to target additional simulations to the most informative regions of parameter space, echoing work on active experimental design for biological network discovery [89]. Model-informed reinforcement-learning frameworks for precision dosing and adaptive therapy already instantiate this pattern *in silico*, using mechanistic simulators as environments while constraining exploration via prior bounds, safety constraints, and periodic refitting to new clinical data [35,36,79].

Across these methods, biological relevance depends on explicit constraints and multi-level validation. Practical implementations combine quantitative metrics for synthetic image and trajectory quality, expert review of simulated morphologies and progression patterns, and evaluation of how synthetic augmentation affects downstream predictive tasks [37,38,49]. Rather than treating synthetic data as valid by construction, parameter-space exploration is best viewed as an iterative hypothesis-generation process in which candidate parameter ensembles are repeatedly confronted with independent real data. Section 4 returns to how formal V&V frameworks can structure this process in clinically meaningful contexts of use.

### 3.3. Computational considerations

Realizing the forward, backward, and closed loops at useful scales requires coordinating three main computational workloads: virtual-tissue simulations, high-volume imaging and feature-extraction pipelines, and training and deployment of AI models. Existing simulation and deployment studies provide a practical envelope for what is currently feasible.

On the simulation side, CompuCell3D routinely handles tens to hundreds of thousands of cells on lattices up to 1024^3^ voxels on a single processor [10], while Cells in Silico extends lattice-based models to ~10^6^ cells and 1000^3^ domains on thousands of CPU cores [92]. The Neural Tissue Simulator and GPU-centric frameworks such as Gell and SimuCell3D demonstrate that millions of cells and billions of synapses can be simulated on leadership-class systems or single high-end GPUs [93–95]. These results imply that ensembles of tissue-scale simulations at histologically relevant resolution are within reach for institutional clusters and cloud resources.

As a concrete scale, a 0.5-mm cube of tissue discretized at 2 μm yields a lattice of roughly 250^3^ (~1.6 × 10^7^) voxels and on the order of 10^5^–3 × 10^5^ cells—one to two orders of magnitude below the largest demonstrations in Cells in Silico or Gell. With center-based or Cellular-Potts agents, this allows hundreds of trajectories to be simulated on modest clusters; even for fully shape-resolved deformable cells, recent benchmarks show that growth from a single cell to ~1.2 × 10^5^ cells in such domains is achievable within about a day on an 8-core CPU [94]. In practice, model complexity, I/O patterns, and required ensemble size, rather than raw hardware limits, dominate feasibility.

Data handling often becomes a bottleneck. Whole-slide imaging and volume EM routinely generate terabyte-scale datasets, requiring streaming, compression, and visualization strategies tailored to limited memory bandwidth and storage [96–98]. For hybrid virtual-tissue–AI systems this argues for co-designing simulation outputs and learning pipelines—for example, tiling 3D fields into patches, caching derived features, and materializing only those quantities needed for downstream tasks.

Resource-optimization strategies therefore matter as much as peak FLOPs. GPU-accelerated cardiac and tissue simulations show one-to two-order-of-magnitude speed-ups relative to CPU-only codes [99,100], while systems like Gell demonstrate that simulations with tens of millions of cells can run on a single high-end workstation [93]. A pragmatic deployment pattern is tiered: large ensemble generation and method development on shared clusters or cloud GPUs, with smaller calibration runs and updates for individual patients on local workstations. Matching spatial and temporal resolution to the clinical question, as discussed in Section 2.5, further keeps costs aligned with context of use.

Equally important is integration into clinical workflows. Studies of AI implementation in hospitals emphasize the “invisible” informatics work required to maintain data quality and align systems with practice patterns [101]. Successful deployments in radiology rely on modular services that plug into PACS, routing engines, and monitoring dashboards and conform to standards such as DICOM and HL7/IHE profiles [102–106]. For virtual-tissue-informed AI, leveraging these architectures will be essential so that synthetic-augmented predictions appear in familiar reporting environments rather than as standalone research tools.

Cloud computing offers elastic access to CPU and GPU resources with pay-as-you-go pricing, and early experience in radiology suggests potential economic and environmental benefits alongside new challenges around data transfer, security, and regulatory compliance [107,108]. Overall, current evidence indicates that the computational demands of the forward, backward, and closed loops are manageable when virtual-tissue models, AI workloads, and clinical integration are co-designed around clearly specified contexts of use and embedded within the validation and governance frameworks discussed in Section 4.

## 4. Validation, bias, and governance for hybrid synthetic-data systems

### 4.1. Risk-informed credibility and context of use

Regulatory agencies already treat computational models as regulated evidence rather than generic research tools. For physics-based medical-device models, ASME V&V 40–2018 and the 2023 FDA guidance on computational modeling and simulation adopt a risk-informed framework: verification, validation, and uncertainty quantification scale with how strongly a model influences a decision and with the consequences of being wrong [38,109]. Both documents emphasize clearly defined questions of interest and contexts of use, as well as structured reporting of credibility evidence. Industrial SOPs that implement V&V 40 translate these ideas into practical workflows graded risk maps linking model influence and decision consequence to credibility goals for verification, validation, and applicability factors.

In drug development, the draft ICH M15 guideline on Model-Informed Drug Development (MIDD) and EMA guidance on PBPK and related mechanistic models extend the same principles to pharmacology: model risk, regulatory role, and decision impact determine how models are planned, evaluated, and documented [110]. Outside biomedicine, the U.S. Environmental Protection Agency’s guidance on environmental models similarly stresses transparent problem formulation, explicit assumptions, and integrated uncertainty and sensitivity analysis. Across these domains, a shared template emerges: fit-for-purpose evaluation, risk-proportional evidence, and traceable documentation.

Domain-specific initiatives push these ideas closer to *in silico* medicine. Good Simulation Practice (GSP), proposed by the Avicenna Alliance, VPH Institute, and the In Silico World project, generalizes V&V 40-style risk-informed credibility to a wide range of biomedical simulations and provides best-practice recommendations for development, evaluation, and reporting, co-developed with regulatory input [37]. In parallel, AHRQ’s Synthetic Healthcare Database for Research (SyH-DR) treats synthetic data as governed infrastructure, with detailed documentation of sampling, synthetization methods, known limitations, and access conditions [111]. Together, these examples show that context of use, model risk, and explicit documentation of limitations are already central to regulatory thinking about computational models and synthetic data. What is still missing is guidance tailored to hybrid systems in which virtual tissues generate synthetic data for AI models and AI, in turn, refines mechanistic models via iterative feedback. Section 4.2 outlines what multi-layer validation might look like for such systems.

### 4.2. Multi-layer validation: From mechanism to workflow

Credibility for hybrid virtual-tissue–AI systems cannot be assessed as a monolith. We treat these systems as a four-layer stack: (i) a mechanistic core, (ii) synthetic data products, (iii) AI components, and (iv) the clinical or operational workflow in which they are embedded [47,67, 109,111,112]. The integrity of this stack relies on a “chain of custody”: machine-readable documentation that links the generating mechanism to the downstream AI decision.

#### Mechanistic model layer.

The virtual-tissue or digital-twin core must satisfy conventional verification and validation criteria, but credibility also depends on transparency and sustainability. Community standards coordinated by the COMBINE initiative—such as SBML for model structure [113] and SED-ML for simulation experiments [114] — provide the necessary infrastructure to encode model equations, parameters, and simulation protocols in a portable, machine-readable form. When bundled into COMBINE archives [115], these formats do more than describe the biology; they serve as the immutable “manufacturing specification” for the synthetic data. This ensures that every synthetic dataset can be traced back to a specific model version and parameter set, supporting both reproducibility and later audits of AI behavior.

#### Synthetic-data layer.

Synthetic outputs must be evaluated as data products rather than assumed valid by construction. At minimum, reports should quantify (i) distributional similarity to real data (fidelity), (ii) performance of downstream models trained on synthetic versus real data (utility), and (iii) privacy risk [1–4,116–118]. Recent frameworks emphasize multi-dimensional evaluation of synthetic health data along these axes [39,116,119]. Analogous to public resources like AHRQ’s SyH-DR (which maintains linkability for governance), virtual-tissue-driven synthetic cohorts should use stable identifiers that connect each synthetic record back to its generating COMBINE archive and its reports. This allows a user to identify exactly which mechanistic hypothesis generated a specific outlier or artifact.

#### AI-component layer.

AI models consuming hybrid real–synthetic data should be evaluated with explicit stratification by data source and clinically relevant subgroups [1–4,19,31,116–118,120–122]. Models trained on augmented datasets must be tested on real-only external cohorts to detect artefacts introduced by synthetic augmentation. Because of the links established between the mechanistic model and the synthetic-data, we can employ explainable-AI (XAI) techniques more effectively [123]. XAI should verify that decision boundaries rely on biologically plausible features rather than quirks of the synthetic generator [41,66,108,124]. If an AI component fails, the “chain of custody” allows developers to debug the underlying mechanistic assumptions rather than just treating the AI as a black box.

#### System and feedback layer.

Finally, credibility must be assessed for the full feedback loop between the digital twin and the patient. As new data arrive, they recalibrate the mechanistic core, which in turn alters the synthetic data used to retrain the AI. Regulatory frameworks (FDA’s risk-informed guidance, Good Simulation Practice (GSP), ICH M15, EMA) highlight three critical credibility factors for this loop [37,38,110]. First, relative vs. absolute accuracy. GSP and MIDD emphasize that for many clinical questions (e.g., “will tumor burden shrink?”), capturing the *direction* of change is more critical than absolute values. Validation metrics must align with this specific Question of Interest. Second, physiological plausibility. Re-fitting to new patient data must keep parameters within biologically interpretable ranges. Large, unexplained shifts should trigger a low-confidence flag and model review rather than forced fit [109]—a safety valve grounded in mechanistic understanding. Third, timeliness as a validity metric. GSP frames “performance” to include timing. If infrastructure limitations delay recalibration beyond the clinical decision window (e.g., the next chemotherapy cycle), the system is invalid for that context of use, regardless of its accuracy [37].

**Fig. 2.**
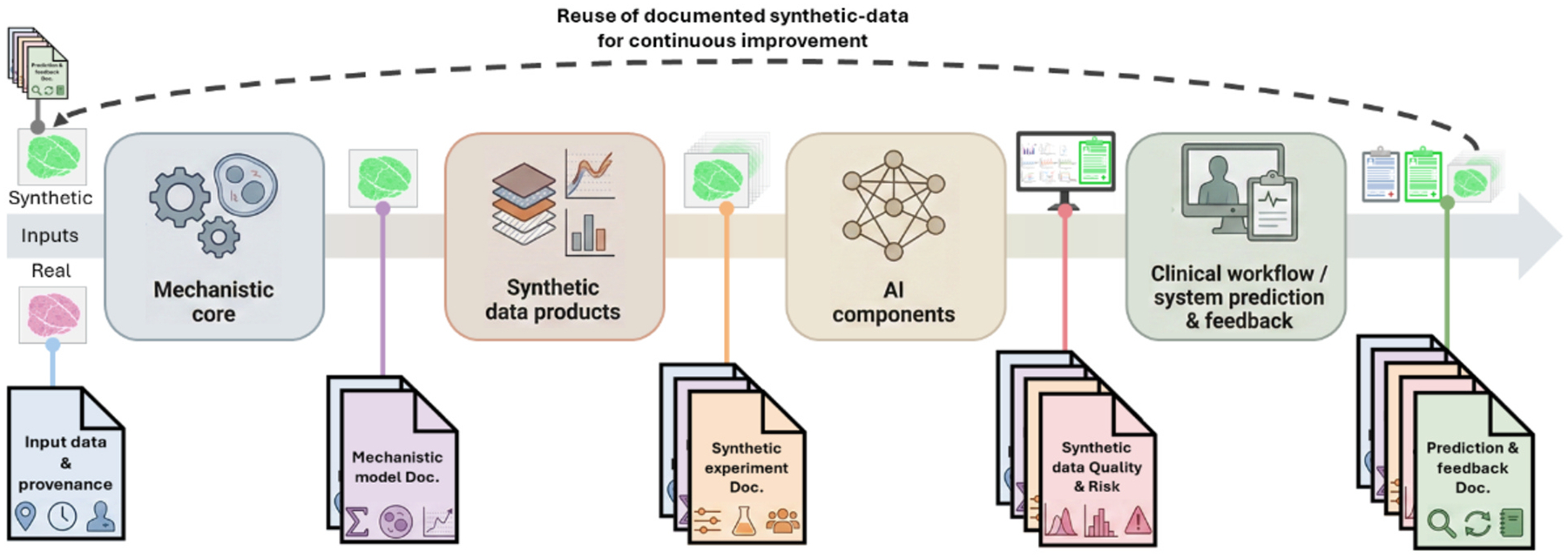
Documentation stack for hybrid mechanistic–synthetic–AI. Data tiles moving left-to-right illustrate that each layer adds documentation, turning synthetic data into an auditable object that links downstream AI behavior to upstream mechanistic hypotheses. The dashed arc indicates the closed feedback loop: as new outcomes arrive, prediction-and-feedback documentation guides recalibration of the mechanistic core and synthetic experiments, and synthetic cohorts from one cycle can re-enter as documented inputs in the next, enabling learning from synthetic data and more transparent AI. Created in BioRender. Vanin, J. (2025) https://BioRender.com/msregew.

Across all four layers, a “shortcoming” is defined relative to the Context of Use. A simplified virtual tissue may be inadequate for generating realistic histology tiles for segmentation training, yet entirely appropriate for simulating longitudinal responses at the cohort level. Credibility, therefore, requires sufficient fidelity in the dimensions that matter for the downstream decision problem, not maximal realism in every aspect [41,47,67,109,111,112]. Fig. 2 summarizes the documentation packages that accompany each phase of the hybrid mechanistic–synthetic–AI system. Documentation is updated iteratively as models are recalibrated, new synthetic cohorts are generated, and AI components are retrained. The rightmost package (system prediction and feedback) feeds backward into the others, so that discrepancies between predictions and real outcomes can be traced to specific assumptions in the input data, mechanistic model, or synthetic generation protocol.

### 4.3. Bias, drift, and provenance in synthetic data

Bias, distributional drift, and incomplete provenance are long-standing concerns in AI and modeling, but hybrid virtual-tissue–AI systems introduce additional channels through which they can arise and be amplified. Synthetic data can mitigate some biases—for example, by up-weighting rare phenotypes—but can also reproduce or worsen existing imbalances if their generation and use are not carefully controlled.

#### Bias and fairness.

Recent reviews of synthetic healthcare data show that generative models often reproduce demographic and clinical skews present in source data, and that naïve augmentation can degrade performance for minority subgroups even when overall metrics improve [1–3,15]. New frameworks therefore propose multi-dimensional evaluation of synthetic data along fidelity, utility, privacy, and fairness axes [39,116,119]. Warnecke et al. (2025) explicitly benchmark the fairness–utility trade-offs of several tabular synthesizers, showing that different algorithms can have markedly different impacts on subgroup performance even at similar global utility [117].

For virtual-tissue-informed synthetic cohorts, this implies that (i) bias assessment should begin with the real calibration datasets; (ii) parameter-sampling strategies should explicitly control subgroup representation (distinguishing between “reference” synthetic populations and targeted oversampling for training); (iii) synthetic data should be evaluated for subgroup-specific fidelity and fairness, not only global similarity; and (iv) any fairness-enhancing interventions (e.g., reweighting, fairness-aware synthesizers) should be documented as part of the dataset’s context of use.

#### Drift and model–data feedback loops.

Hybrid systems are also vulnerable to classical data drift (changes in practice patterns, populations, or disease biology) and to model-induced drift, where models are repeatedly trained on their own outputs or on data shaped by earlier predictions. Recent work on “model collapse” shows that training generative models solely on synthetic data from previous generations erodes coverage of distributional tails and minority patterns [118,120]. Kazdan et al. (2025) demonstrate that “replace” workflows, where real data are iteratively replaced by synthetic surrogates, reliably produce collapse across diverse tasks, whereas “accumulate” workflows that retain real data can maintain stable performance [121].

These are general statistical phenomena, not specific to language models, and apply directly to virtual histology generators and stochastic parameter-sampling schemes. In practice, they argue for (i) retaining a persistent anchor set of real examples that is never used for training, (ii) training under accumulate or accumulate–subsample regimes with predefined caps on the synthetic-to-real ratio, and (iii) using mixture workflows in which synthetic data supplement rather than replace real data [120,121]. At the same time, recent methods such as Self-IMproving diffusion models with Synthetic data (SIMS) show that carefully designed self-training strategies can use synthetic data to *reduce* bias and resist degradation by treating self-generated samples as negative guidance [122]. Ferrara’s analysis of the “butterfly effect” in AI systems further underscores how small initial skews can be amplified through feedback loops, motivating continuous monitoring of both input distributions and subgroup performance with triggers for recalibration or rollback [125].

#### Provenance, documentation, and governance.

Provenance—the ability to trace how synthetic data and models were created, from which sources, and for what purposes—is essential for scientific credibility and governance. The AHRQ–NIMHD panel’s principles for mitigating algorithmic bias in health care call for transparency about training data, development processes, evaluation methods, and continuous equity monitoring across the algorithm life cycle [126]. Reviews of synthetic health data similarly argue that responsible use requires auditable quality and bias assessments and a “chain of custody” that tracks synthetic data from generation through downstream modeling and deployment [40,127]. From the broader AI perspective, Gebru et al.’s “datasheets for datasets” provide a concrete template for documenting dataset motivation, composition, collection, recommended uses, and known risks [128].

For virtual-tissue-informed synthetic cohorts, an analogous practice would be to accompany each dataset with a synthetic data card that records at minimum: (i) the real datasets and time windows used for calibration; (ii) the versioned virtual-tissue and generative models that produced the synthetic samples; (iii) the mixture of synthetic and real data used in training downstream AI components and the contribution of each to performance; (iv) evaluation metrics for fidelity, utility, privacy, and fairness; and (v) intended contexts of use and known limitations. Making these artefacts machine-readable and linking them to model cards for the AI components would help clinicians, regulators, and researchers understand not only *what* a hybrid system predicts, but *how* those predictions depend on particular combinations of real and synthetic data and mechanistic versus learned structure.

Treating synthetic data and hybrid models as first-class, documented artefacts has several benefits. It enables reproducible science by allowing other groups to regenerate virtual cohorts under similar assumptions; it supports regulatory and institutional review by making explicit the assumptions and transformations from original measurements to synthetic datasets and AI predictions; and it facilitates post-deployment surveillance by allowing auditors to reconstruct which model and dataset versions underpinned specific decisions. Integrating bias and drift monitoring with such provenance tools—datasheets, synthetic data cards, and model cards—should be considered a core design requirement for virtual-tissue–AI systems rather than an optional post hoc add-on.

## 5. Discussion and conclusions

Taken together, our analysis suggests that synthetic data in medicine is not something to fear in itself, but something to govern. What should worry us are hybrid pipelines in which synthetic cohorts are generated without transparent mechanistic assumptions, mixed with real data without provenance, and deployed without risk-informed validation or monitoring for drift and bias. The central claim of this paper is that virtual tissues, digital twins, and AI/ML should be treated as parts of a single, coupled system whose credibility depends on how well the forward loop (mechanistic → synthetic → AI), backward loop (AI → mechanism), and closed loop (patient-anchored twin) are coordinated and constrained.

Conceptually, we have argued for two complementary structuring principles. The first is a loop-based view in which virtual tissues serve as mechanistic engines for synthetic cohorts, AI components act as surrogates and inference engines, and digital twins close the loop with individual patients or cohorts. The second is a four-layer credibility stack—mechanistic core, synthetic-data products, AI models, and clinical workflow—linked by a machine-readable chain of custody. This stack allows existing frameworks such as ASME V&V 40, Good Simulation Practice, MIDD, and emerging synthetic-data evaluation metrics to be mapped onto hybrid systems in a way that is proportional to context of use and decision risk.

In practical terms, we see three near-term priorities. First, develop and share reference virtual-tissue models and synthetic cohorts (with COMBINE-style archives and synthetic-data cards) that can serve as common benchmarks for hybrid architectures. Second, adopt mixture training and evaluation strategies in which synthetic data augment rather than replace real-world data, with explicit monitoring for collapse, drift, and subgroup performance. Third, embed early hybrid systems in well-defined, narrow clinical use cases—such as specific cancer indications or imaging-guided therapy decisions—where prospective, V&V 40–style evaluation and workflow integration can be realistically achieved. Longer term, we anticipate fully closed-loop digital twins in selected domains, supported by infrastructure for continuous recalibration, explainability, and regulatory oversight.

Finally, the stakes are highest precisely where data are scarcest: rare diseases, atypical phenotypes, and under-represented populations. Here, virtual-tissue-driven synthetic data can help expand the space of plausible trajectories and treatment responses, but only if fairness and subgroup-specific fidelity are treated as first-class evaluation targets. Progress will therefore depend on sustained collaboration between modelers, clinicians, data scientists, and regulators, as well as on mundane but essential investments in data curation and informatics. If those conditions are met, hybrid mechanistic–AI systems can move synthetic data from an object of suspicion to a trustworthy, auditable extension of our measurement capabilities—one that both respects patient privacy and deepens biological insight in the service of precision medicine.

## Figures and Tables

**Table 1:** Toolkit for parameter-space exploration in hybrid virtual-tissue–AI systems. Methods are grouped by their primary role in the forward loop (mechanistic → synthetic data → AI), backward loop (AI → mechanistic core), or cross-cutting support for both.

Method	Loop role	What it offers for parameter exploration	Illustrative references*
Manifold learning/dimensionality reduction	Forward	Identifies low-dimensional embeddings of high-dimensional dynamics, enabling efficient sampling of parameter regimes while preserving key behaviors and trajectories.	Parameter reduction [33]; Parameter estimation [82].
Virtual staining/generative models for histology	Forward	Conditioned realistic, spatially resolved synthetic histology; supports augmentation and stress-testing of image-based AI.	Generative pathology workflows [32]; Virtual staining [83]; Synthetic pathology evaluation [49].
Simulation-based inference (SBI)	Backward	Approximate parameter posteriors given observed imaging/biomarker trajectories; support construction of virtual patients consistent with model structure and measured variability.	SBI methods [68,84]; SBI Virtual patients [69].
Autoencoders/variational autoencoders (VAEs)	Backward	Compress high-dimensional simulation outputs into latent spaces and learn correspondences between observed patterns and regions of parameter space.	Autoencoder for histopathology [71]; Feature extraction from tissue [72].
Bayesian networks	Both	Encode prior mechanistic structure and uncertainty; link heterogeneous observables to latent mechanistic states or parameter clusters; make conditional dependencies explicit and inspectable.	BN primer in computational biology [85]; BNs for bio. network inference [86].
Symbolic regression (e.g., PySR, LogicSR)	Backward/interpretability	Discover explicit human-readable equations mapping experimental features to parameters and/or rules.	PySR [87]; LogicSR [88].
Active learning/model-informed RL	Efficiency/closed loop	Uses uncertainty over class labels, parameter posteriors, or policy performance to target simulations to the most informative regions of parameter space.	Network discovery [89]; model-informed RL for cancer therapy and dosing [35,36, 79].
